# Early results of high tibial osteotomy versus combined arthroscopic surgery

**DOI:** 10.3389/fsurg.2024.1325483

**Published:** 2024-01-16

**Authors:** Zhenbin Zhang, Zhaolong Yan, Gongteng Wang, Wenqi Zhang, Guangxing Li, Xufeng Wang, Huaqiang Sun, Shufeng Li

**Affiliations:** ^1^ Shandong Key Laboratory of Rheumatic Disease and Translational Medicine, Department of Orthopedic Surgery, The First Affiliated Hospital of Shandong First Medical University & Shandong Provincial Qianfoshan Hospital, Jinan, Shandong, China; ^2^ Department of Orthopedic Surgery, First Clinical Medical College of Shandong University of Traditional Chinese Medicine, Jinan, Shandong, China

**Keywords:** medical knee osteoarthritis, unicompartmental osteoarthritis, high tibial osteotomy, arthroscopy, surgical effect, influencing factors

## Abstract

**Objective:**

To investigate the early effect of high tibial osteotomy (HTO) compared with combined arthroscopic surgery.

**Methods:**

A retrospective study was conducted on patients who underwent HTO at The First Affiliated Hospital of Shandong First Medical University from January 2018 to January 2022. 138 patients (163 knees) with knee osteoarthritis (KOA) treated with HTO were selected. The medial proximal tibial angle (MPTA), joint line convergence angle (JLCA), femoral tibial angle (FTA), hip-knee-ankle (HKA) angle, weight-bearing line (WBL) ratio of the knee joint, opening gap, opening angle, American Knee Society score (KSS), US Hospital for Special Surgery (HSS) score, and Western Ontario and McMaster Universities Osteoarthritis Index (WOMAC) score were measured to determine the different effects between HTO and HTO combined with arthroscopic by logistic regression analysis.

**Results:**

Patients with HTO combined with arthroscopic surgery have improved functional scores as well as imaging perspectives compared to preoperative. By multivariate logistic analysis, it was concluded that arthroscopic surgery and gender are influential factors in the outcome of HTO surgery. The postoperative KSS score was 2.702 times more likely to be classified as excellent in the HTO combined with arthroscopy group than in the HTO group [Exp (β) = 2.702, 95% CI (1.049–6.961), *P* = 0.039]; the postoperative KSS score was 0.349 times more likely to be classified as excellent in women than in men [Exp (β) = 0.349, 95% CI (0.138–0.883), *P* = 0.026].

**Conclusion:**

Better results with HTO combined with arthroscopic surgery. HTO combined with arthroscopy is a better choice in the surgical treatment of KOA.

## Introduction

Osteoarthritis is a joint disease of great concern in the world today and is characterized by synovial hyperplasia, articular cartilage degeneration, and the formation of osteophytes, which can eventually lead to pain, joint loss, and disability (1, 2). One part of the pathogenesis of knee osteoarthritis (KOA) is related to the natural degeneration of articular cartilage, and the other part is affected by the biomechanics of the lower extremities (3). In patients with internal and external knee deformity, lower extremity force line abnormalities have a significant effect on KOA progression by increasing the stress load on the medial and lateral compartments, further causing articular cartilage and subchondral bone damage (4). Knee varus deformity has been found to affect the progression of KOA more significantly than valgus deformity (5). Degenerative arthritis has many treatment options. Surgical treatments include arthroscopic surgery, corrective osteotomies and knee replacements, while conservative treatments include medications and physical therapy. It is crucial to choose the right course of action based on the patient's age, degree of activity, arrangement of their lower extremities, and body mass index (6). Therefore, the treatment of KOA for symptomatic patients with knee varus and valgus deformities through surgery to improve lower limb biomechanics has a positive effect, and high tibial osteotomy (HTO) is widely used. HTO was first reported by Jackson et al. in 1958 for the treatment of medial compartment osteoarthritis of the knee, and its efficacy has been demonstrated by a large number of clinical studies (7–9) HTO is mainly used to reduce unilateral compartment load by correcting lower limb alignment, thereby relieving pain, delaying knee replacement time, and restoring high levels of patient activity (10). HTO combined with arthroscopic surgery in one stage can treat intra-articular combined injuries and significantly improve short-term clinical symptoms. In this paper, we retrospectively analyze the clinical data of patients applying HTO with and without combined arthroscopic surgery to investigate whether arthroscopic surgery is an independent influencing factor on the outcome of HTO surgery.

## Materials and methods

### General information

The study subjects were patients who underwent medial open HTO for medical KOA at The First Affiliated Hospital of Shandong First Medical University from January 2018 to January 2022. The inclusion criteria were (1) lesions involving only the medial compartment of the knee, cartilage degeneration into Kellgren–Lawrence II, III, and other compartment cartilage degeneration less than Kellgren–Lawrence II, (2) intact anterior and posterior cruciate ligament with stable knee, and (3) aged ≤70 years with high requirements for postoperative activities. The exclusion criteria were (1) patients with infections and rheumatoid arthritis, (2) patients with a history of knee trauma, (3) patients with severe cardiovascular and cerebrovascular diseases, (4) patients with severe osteoporosis, (5) patients with a history of local treatment, and (6) patients with nerve, muscle, or connective tissue diseases.

### Surgical methods

The surgeries were performed by two physicians with senior titles in the Department of Arthroscopy of The First Affiliated Hospital of Shandong First Medical University, and were divided into HTO group and HTO combined arthroscopy group according to the different surgical habits of the two surgeons (one surgeon only did HTO and one only did HTO combined arthroscopy).

Arthroscopy treatment: (1) Microscopic exploration involved the arthroscopic exploration of the medial and lateral knee joint standard approach, arthroscopic exploration of the medial and lateral compartment and patellofemoral articular cartilage degeneration, and recording to provide some reference for alignment correction position. (2) Microscopic treatment: According to the lesion, the injured meniscus was trimmed, and the narrow intercondylar notch was formed. Then, the stripped cartilage and loose bodies were removed, and the synovial folds causing intra-articular entrapment impingement were removed. Microfractures were performed for a small range of full-thickness cartilage defects, and suturing was performed after adequate lavage.

HTO treatment: HTO was performed after the arthroscopy treatment. A 6–8-cm straight incision was made in the medial aspect of the proximal tibia, and the proximal flat joint line was exposed layer by layer until the medial aspect of the tibia and the posterior tibial crest were exposed. Two 2.0 cm Kirschner wires were inserted in parallel at 3.5 cm from the upper edge of the tibia and 1.5 cm from the upper edge of the tibia along the outer edge of the tibia to penetrate the contralateral cortex. The Kirschner wire plane remained consistent with the posterior slope of the tibial plateau. Horizontal resection surface: Resection was performed close to the distal ends of the two Kirschner wires, leaving approximately 1 cm from the lateral cortex as a hinge point. Coronal osteotomy plane: The patellar tendon insertion was clamped at 110° to the horizontal osteotomy plane starting above the patellar tendon insertion to ensure that the patellar tendon insertion remained attached to the distal side of the osteotomy after osteotomy correction, then slowly distracted through the osteotome or spreader to reach the desired distraction angle for the preoperative plan. The alignment was determined by alignment rod and fluoroscopy and fixed with a TomoFix locking plate (Johnson & Johnson), and different osteotomy gap treatment methods were selected according to the distraction angle and bone quality.

### Radiographic measurements and functional score follow-up

**Figure 1:**
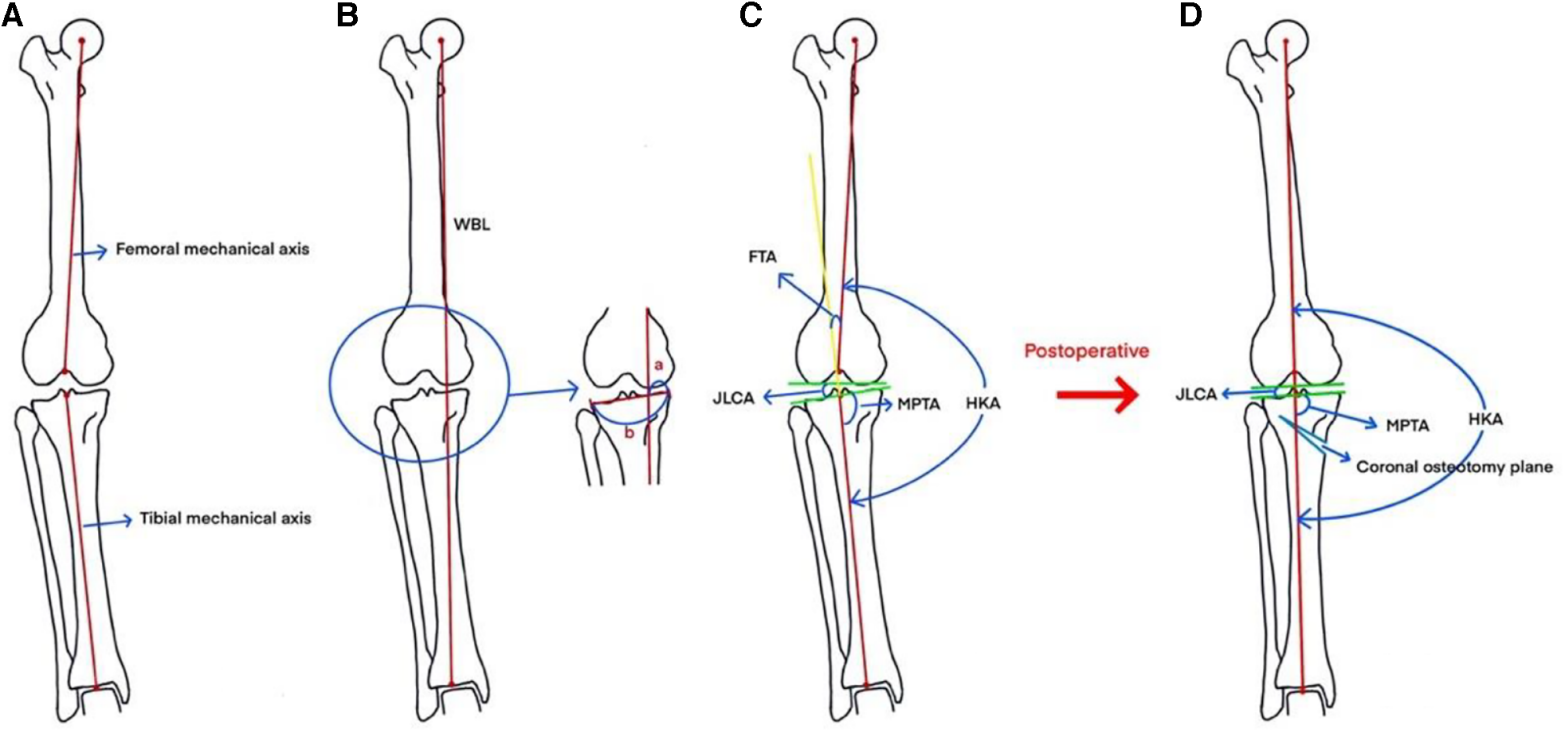
(**A**) Femoral mechanical axis: the line connecting the center of the femur and the apex of the intercondylar fossa of the femur. (**B**) Measurement of WBL: The line connecting the center of the femoral head and the center of the ankle joint is the WBL. (**C**) Measurement of WBL ratio: a is the distance between the medial tibial cortex and the force line along the proximal direction of the tibia, b is the distance between the medial and lateral tibial cortex, and the ratio of a and b is the WBL ratio of the knee. Measurement of FTA: The angle between the extension of the tibial mechanical axis and the femoral mechanical axis is the tibiofemoral angle. The angle between the extension of the mechanical axis of the tibia and the mechanical axis of the femur is the tibiofemoral angle. Measurement of HKA: the angle between the mechanical axis of the tibia and the mechanical axis of the femur is the hip-knee-ankle angle. Measurement of JLCA: the angle formed between the relative joint running direction lines of the same joint. Measurement of MPTA: draw the running direction line of the proximal tibial joint, draw a line on the tibio-knee line from the center point of the ankle joint to the center point of the knee joint The angle between the two lines is the MPTA. (**D**) The force line and imaging angle of the lower extremity is improved after HTO.

Radiographic measurements were performed by an independent observer using standard preoperative and 12-month postoperative full-length anteroposterior radiographs of both lower extremities obtained during clinical follow-ups. The imaging parameters included MPTA, JLCA, HKA angle, FTA, WBL ratio of the knee joint, postoperative opening gap, and postoperative opening angles (Figure 1). Functional score follow-up was obtained by an independent observer at least 12 months after surgery in the form of an outpatient consultation or telephone follow-up. Functional scores include HSS, KSS, WOMAC. The postoperative KSS scores were divided into two clinically relevant categories according to the results: excellent ≥ 85 points and poor < 85 points (11, 12).

### Statistical methods

The continuous variables, such as preoperative and postoperative radiographic parameters and various scores of patients in the HTO combined with arthroscopy group, were tested for a normal distribution using the Shapiro–Wilk test. A paired t-test was performed for the measurement of data meeting a normal distribution, and the results were expressed as the mean ± standard deviation. A paired Wilcoxon signed-rank test was used for the measurement of data with a skewed distribution, and the results were expressed as the median (interquartile range) [M (IQR)]. Patients were screened univariately for general demographic data, presence or absence of joint arthroscopy, functional scores, and various imaging parameters. If the independent variable *P* < 0.05 for univariate screening appeared in the analysis results, the variable was included in the multivariate logistic regression analysis and the difference was considered statistically significant at *P* < 0.05. All statistical analyses were performed using IBM SPSS Statistics 26.

## Results

### Follow-up

During this period, 148 patients received HTO in 173 knees. Five patients were lost during follow-up, five patients had incomplete imaging data, and 163 limbs of 138 patients were finally included in this group. Patients were divided into two groups on the basis of the presence or absence of joint arthroscopy (HTO group and HTO combined with arthroscopy group). A total of 103 patients (121 knees) were included in the HTO group, among whom 18 were bilateral and all were staged operations, with 47 (45.6%) males and 56 (54.4%) females. Thirty-five patients (42 knees) were included in the HTO combined with arthroscopy group, among whom seven were bilateral and four were staged operations. Three were concurrent operations, among whom 21 (60%) were males and 14 (40%) were females. Comparison of patients' baseline data, the difference was not statistically significant (*P* > 0.05) and was comparable (Table 1).

**Table 1:** Comparison of the general data of 138 patients (163 knees) with osteoarthritis of the knee who underwent medial open HTO.

Parameter	HTO group	HTO combined with arthroscopy group	*χ*^2^/t/Z values	*P* values
Number of patients (knees)	103 (121)	35 (42)		
Age [M(IQR), year]	54 (6)	53 (6)	−1.412	0.158
Height [M(IQR), meter]	1.65 (0.12)	1.62 (0.10)	−0.044	0.965
Weight [M(IQR), kg]	75.0 (20.0)	72.0 (12.0)	−1.466	0.143
BMI ($\bar{X}\pm S$, kg/m^2^)	28.04 ± 0.30	27.09 ± 0.53	1.589	0.114
Gender [case (%)]				
Male	47 (45.6%)	21 (60%)	1.285	0.257
Female	56 (54.4%)	14 (40%)		

HTO, high tibial osteotomy; BMI, body mass index.

### Radiographic improvement

**Figure 2:**
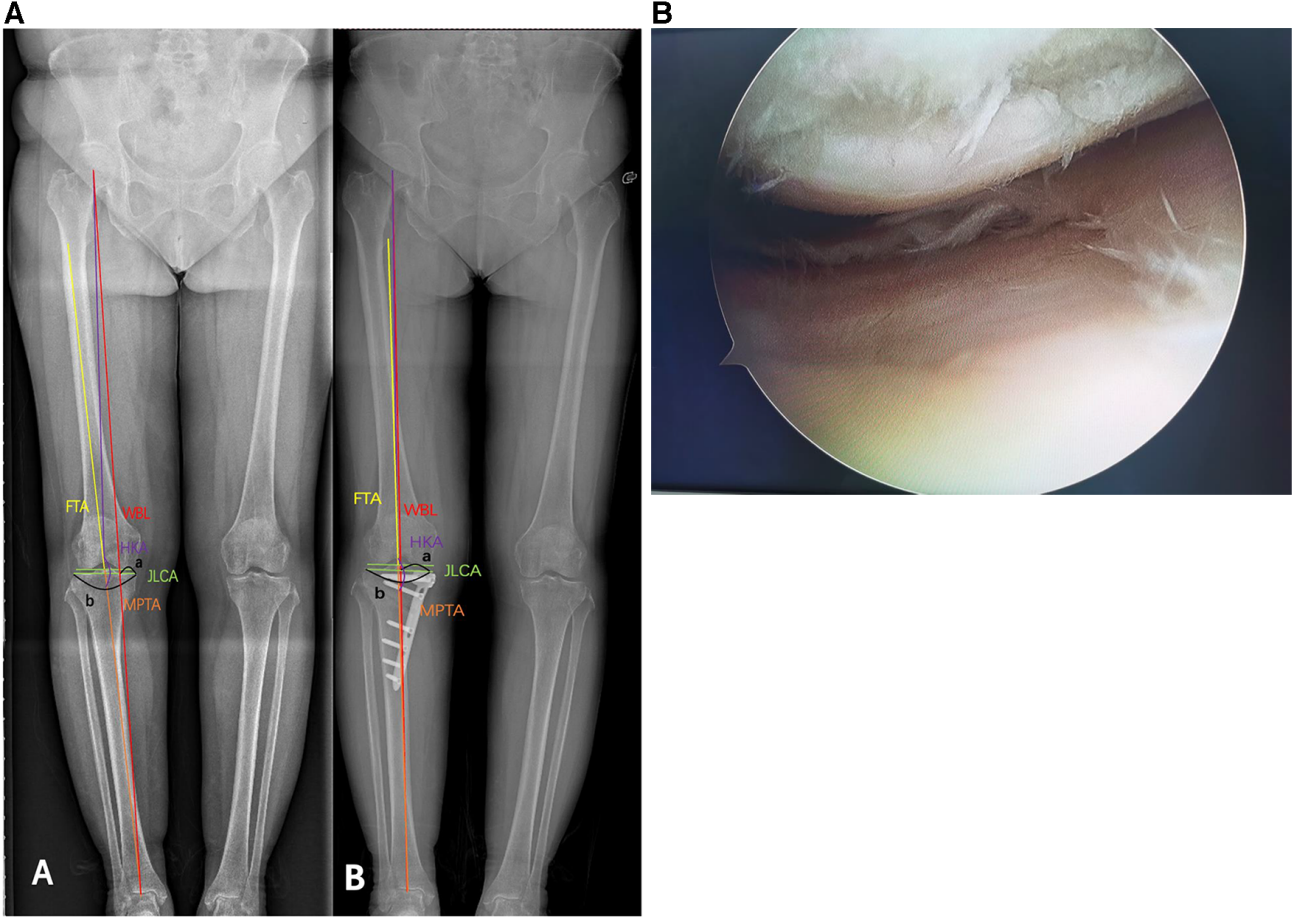
Images from a 57-year-old female patient showing (**A**) preoperative and postoperative x-ray comparison (“**A**” is preoperative, “**B**” is postoperative). Preoperative WBL ratio of the knee joint: 25.39%, and postoperative WBL ratio of the knee joint: 46.80%. After the operation, the radiographic parameters were significantly improved. (**B**) Preoperative examination showed McMurray (+), and intraoperative arthroscopy showed subchondral bone exposure of the medial femoral condyle and radial tears from the body of the medial meniscus to the posterior horn. Postoperative follow-up pain was significantly relieved, McMurray (–).

The postoperative imaging indicators in the two groups were significantly improved compared with those before surgery (Tables 2, 3). Postoperative MPTA increased compared with that before surgery, JLCA decreased, FTA decreased, HKA increased, and WBL% increased. The differences were statistically significant (Figure 2).

**Table 2:** Pre- and post-operative radiographic performance of patients with osteoarthritis of the knee in the HTO group.

Parameters	Preoperative values	Postoperative values	Statistical values	*P* values
MPTA [M(IQR), °]	84 (3)	93 (4)	Z = −4.463	<0.001
JLCA [M(IQR), °]	3 (3)	3 (2)	Z = −1.969	<0.05
FTA [M(IQR), °]	9 (4)	1 (3)	Z = −4.424	<0.001
HKA [M(IQR), °]	171 (5)	180 (1)	Z = −7.145	<0.001
WBL ratio of the knee joint [M(IQR), %]	14.5% (16.32%)	53.4% (17.76%)	Z = −4.107	<0.001

**Table 3:** Pre- and post-operative radiographic performance of patients with osteoarthritis of the knee in the HTO combined with arthroscopy group.

Parameters	Preoperative values	Postoperative values	Statistical values	*P* values
MPTA [M(IQR), °]	84 (4)	93 (8)	Z = −2.366	<0.05
JLCA [M(IQR), °]	3 (1)	1 (1)	Z = −2.539	<0.05
FTA [M(IQR), °]	6 (6)	2 (5)	Z = −2.313	<0.05
HKA [M(IQR), °]	174 (7)	179 (4)	Z = −2.490	<0.05
WBL ratio of the knee joint [M(IQR), %]	24.9% (22.14%)	53.6% (30.36)	Z = −2.366	<0.05

MPTA, medial proximal angle; JLCA, joint line convergence angle; FTA, femoral tibial angle; HKA, hip-knee-ankle angle; WBL, weight-bearing line ratio of the knee joint.

### Postoperative clinical function comparison

**Figure 3:**
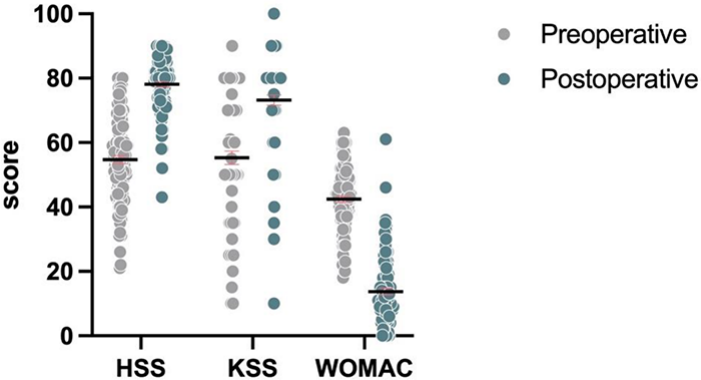
Comparison of preoperative and postoperative HSS, KSS, and WOMAC histograms in the HTO group.

**Figure 4:**
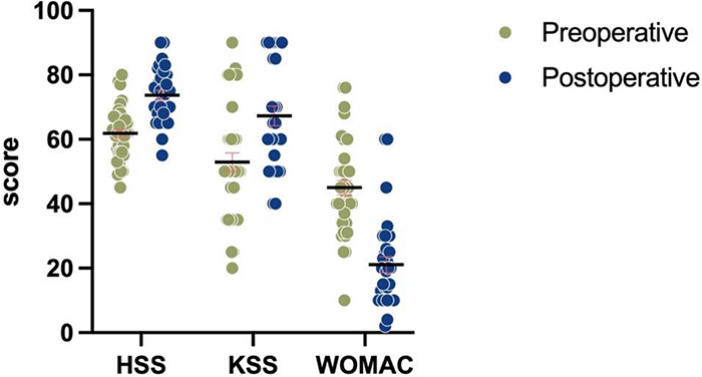
Comparison of preoperative and postoperative HSS, KSS, and WOMAC histograms in the HTO combined with arthroscopy group.

Postoperative HSS scores and KSS scores were significantly higher in both groups compared to preoperative scores, the WOMAC scores decreased, which was significantly different from preoperative (Table 4, Figure 3, Table 5, and Figure 4), and knee function improved.

**Table 4:** Comparison of clinical outcomes of knee joints before and after surgery in patients with osteoarthritis of the knee in the HTO group.

Parameters	Preoperative	Postoperative	Z values	*P* values
HSS [M(IQR), score]	55.0 (18.0)	80.0 (13.0)	−8.783	<0.001
KSS [M(IQR), score]	57.5 (41.0)	80.0 (10.0)	−5.806	<0.001
WOMAC [M(IQR), score]	44.0 (12.0)	11.0 (12.0)	−8.918	<0.001

**Table 5:** Comparison of clinical outcomes of knee joints before and after surgery in patients with osteoarthritis of the knee in the HTO combined with arthroscopy group.

Parameters	Preoperative	Postoperative	Z values	*P* values
HSS [M(IQR), score]	62.0 (10.0)	74.0 (12.0)	−4.686	<0.001
KSS [M(IQR), score]	50.0 (25.0)	60.0 (40.0)	−3.985	<0.001
WOMAC [M(IQR), score]	43.5 (12.0)	20.0 (16.0)	−4.866	<0.001

### Factors influencing the outcome of HTO surgery

Whether HTO was combined with arthroscopy was set as the independent variable “ Arthroscopy”, and the patients' general data (age at surgery, sex, height, weight, and BMI), preoperative functional scores, and various imaging parameters (MPTA, JLCA, FTA, HKA angle, and WBL%, opening gap, opening angle) were screened by single independent variables by factorial analysis. The postoperative KSS score categories (≥85: excellent and <85: not excellent) (11, 12) were included as dependent variables in a binary logistic regression analysis, with a screening *P*-value set at 0.05. The corresponding independent variables were screened as Group (*P* = 0.036) and Gender (*P* = 0.025) (Table 6).

**Table 6:** Binary logistic regression analysis of 138 patients with osteoarthritis of the knee with postoperative KSS score as the dependent variable.

Parameters	β	Wald	OR	95% CI	*P* values
Arthroscopy	0.985	4.388	2.679	1.065–6.734	0.036
Gender	−1.046	5.042	0.351	0.141–0.875	0.025
Age	0.006	0.019	1.006	0.928–1.090	0.891
Height	2.752	1.052	15.680	0.081–3019.758	0.305
Weight	0.003	0.034	1.003	0.970–1.038	0.854
BMI	−0.042	0.431	0.959	0.845–1.088	0.511
Preoperative HSS	0.005	0.082	1.005	0.970–1.041	0.774
Preoperative WOMAC	−0.020	1.084	0.980	0.943–1.018	0.298
Preoperative KSS	0.002	0.043	1.002	0.982–1.023	0.835
Preoperative MPTA	0.108	1.888	1.114	0.955–1.299	0.169
Postoperative MPTA	0.096	0.424	1.101	0.824–1.472	0.515
Preoperative JLCA	−0.001	0.000	0.999	0.765–1.305	0.993
Postoperative JLCA	0.619	2.919	1.857	0.913–3.775	0.088
Preoperative FTA	−0.087	1.521	0.917	0.798–1.053	0.218
Postoperative FTA	0.217	1.429	1.243	0.870–1.774	0.232
Preoperative HKA	0.068	1.310	1.071	0.952–1.204	0.252
Postoperative HKA	−0.274	2.419	0.760	0.538–1.074	0.120
Preoperative WBL%	0.022	1.141	1.022	0.982–1.065	0.286
Postoperative WBL%	0.044	0.630	1.045	0.938–1.164	0.427
Postoperative opening gap	−0.010	0.020	0.990	0.859–1.141	0.887
Postoperative opening angle	−0.011	0.016	0.989	0.838–1.168	0.899

Arthroscopy, Gender was included in the multivariate logistic regression analysis at the same time, the results found statistically significant differences for both Arthroscopy and Gender (Table 7).

**Table 7:** Results of the multiple logistic regression analysis of group and gender.

Parameters	β	Wald	OR	95% CI	*P* values
Arthroscopy	0.994	4.241	2.702	1.049–6.961	0.039
Gender	−1.053	4.945	0.349	0.138–0.883	0.026

The postoperative KSS score was 2.702 times more likely to be classified as excellent in the HTO combined with arthroscopy group than in the HTO group [Exp (β) = 2.702, 95% CI (1.049–6.961), *P* = 0.039]; the postoperative KSS score was 0.349 times more likely to be classified as excellent in women than in men [Exp (β) = 0.349, 95% CI (0.138–0.883), *P* = 0.026].

## Discussion

In this study, we found that both HSS and KSS scores were significantly higher in HTO combined arthroscopic patients postoperatively compared to preoperatively. WOMAC scores decreased in patients after HTO combined arthroscopic surgery compared to preoperative, and patients showed significant improvement in functional knee recovery and imaging parameters. Arthroscopy and gender are independent influences on the outcome of HTO surgery. Most authors have concluded that gender is an independent factor affecting the outcome of HTO, with female patients recovering worse than male patients after surgery (13). Bouguennec (14) concluded that gender is an independent factor affecting the outcome of HTO by performing a statistical analysis of patients who underwent HTO over a 7–10 year follow-up period. A similar conclusion was reached in this study, where women were 0.349 times more likely than men to have an excellent postoperative KSS score. Yoo et al. (15) concluded that medial open HTO combined with arthroscopic surgery is an effective method for treating medial interval KOA and intra-articular lesions with combined inversion deformity. Schuster et al. (16) found that good imaging and clinical results were achieved after performing proximal tibial osteotomy combined with arthroscopic scraping and microfracture, with 94.9% of patients satisfied. In our study, a significant improvement in the overall functional scores of patients after combined arthroscopic surgery with HTO was observed, and all postoperative imaging indices were significantly improved compared with preoperative ones. Thus, similar conclusions were obtained in this study. We believe that the combined arthroscopic surgical approach can shape a damaged meniscus and cartilage, clean up a proliferated synovial membrane, and reduce the inflammatory response by performing arthroscopic microfractures for large cartilage defects in which drilling under the cartilage allows the fracture fat and blood to leak out naturally. The formed blood clot can fill the cartilage defect site, while the mesenchymal stem cells in the bone marrow can effectively repair the cartilage defect under the regulation of the repair mechanism. Pascale et al. (17) prospectively studied 40 patients with medial degenerative arthritis of the knee, divided into two groups: proximal tibial osteotomies with and without arthroscopic microfractures. There were no significant differences between the two groups, but subjective satisfaction was higher in the former during the five-year follow-up. Harris et al. (18) reviewed several publications and concluded that the five-year survival rate was 97.7% for proximal tibial osteotomy combined with arthroscopic surgery and 92.4% for proximal tibial osteotomy alone. Proximal tibial osteotomy combined with arthroscopic surgery was superior to proximal tibial osteotomy alone in terms of survival; however, there were many differences between the studies in terms of surgical technique, follow-up time, inclusion criteria, and severity of medial compartment osteoarthritis. Our study screened the univariate variables as to whether to combine arthroscopy and gender by binary logistic analysis, and then included both in multivariate logistic analysis to confirm, first, that arthroscopy was an independent influencing factor on the outcome of HTO surgery, and second, the postoperative KSS score was 2.702 times more likely to be classified as excellent in the HTO combined with arthroscopy group than in the HTO group. Therefore, our findings add weight to the results described in the above studies. Although the effect of combined arthroscopic surgery for HTO has been confirmed by many experts, and the postoperative influences of HTO have been reported and confirmed (19, 20), the controlled grouping of HTO with or without combined arthroscopy and long-term follow-up retrospective studies of the independent influence of arthroscopy on the surgical outcome of HTO have rarely been reported.

Due to the higher chance of complications and injury to the common peroneal nerve in lateral closed wedge tibial high osteotomy (CWHTO), the main procedure used now is medial open wedge tibial high osteotomy (OWHTO) (21–23). Considering the possible effects of different surgical approaches on postoperative function, for comparison, only patients with OWHTO were collected in this paper.

Since the 1960s, HTO has been used to treat unicompartmental osteoarthritis of the knee (24–26). HTO corrects the weight-bearing axis of the lower extremity by osteotomy or slightly overcorrects to achieve mild knee valgus and changes the position of the force line, thus changing the pressure load of the medial and lateral compartments of the knee, shifting the load of the medial compartment to the normal lateral compartment, effectively reducing the pressure of the medial compartment, stopping cartilage wear, and improving the conditions for cartilage repair (27–29). This effectively addresses biomechanical and structural abnormalities in patients with knee osteoarthritis, improving patients' knee function and quality of life, and slowing the progression of degenerative osteoarthritis (30, 31) as well as delaying or eliminating the need for joint replacement to the greatest extent possible.

There are some limitations to this study. First, the sample size of the combined HTO arthroscopy group was small. Secondly, the surgeons were not the same person, and the results would produce some bias. Thirdly, our study was a retrospective case study, which is a type of study inherently subject to various sources of bias, including selection bias, measurement and assessment bias, and missed visits. Finally, this was a short- to medium-term follow-up study that did not provide long-term follow-up results.

## Conclusion

HTO combined with arthroscopic surgery has better clinical results than HTO surgery alone, and arthroscopy is an independent factor affecting the outcome of HTO surgery. Therefore, it makes sense to combine HTO surgery with arthroscopic surgery.

## Data Availability

The original contributions presented in the study are included in the article/Supplementary Material, further inquiries can be directed to the corresponding author.
